# Evidence on acne therapy*

**DOI:** 10.1590/1516-3180.2013.1313616

**Published:** 2013-06-01

**Authors:** Caroline Sousa Costa, Ediléia Bagatin

**Affiliations:** I MD. Doctoral student in the Postgraduate Program on Internal Medicine and Therapeutics, Escola Paulista de Medicina - Universidade Federal de São Paulo (EPM-Unifesp), and Dermatologist at the STD/AIDS Reference and Training Center, Government of the State of São Paulo, São Paulo, Brazil.; II MD, PhD. Dermatologist and Adjunct Professor, Department of Dermatology, Escola Paulista de Medicina - Universidade Federal de São Paulo (EPM-Unifesp), São Paulo, Brazil.

**Keywords:** Acne vulgaris, Therapeutics, Evidence-based medicine, Anti-bacterial agents, Isotretinoin, Acne vulgar, Terapêutica, Medicina baseada em evidências, Antibacterianos, Isotretinoína

## Abstract

Among the current treatments available for acne vulgaris, many widely practiced options lack support from studies at the best level of scientific evidence. The aim of this narrative review was to present the very latest information on topical and systemic treatments for acne vulgaris. Information from systematic reviews and well-designed clinical trials, obtained through a systematic search of the major medical databases, is emphasized. There are important issues regarding the clinical management of acne that still lack consistent grounding in scientific evidence. Among these are the optimum dose and duration of treatment with oral antibiotics that can be given without inducing bacterial resistance, and the safety of oral isotretinoin.

## RESUMO

Na terapêutica atual da acne vulgar, muitas opções amplamente praticadas carecem de respaldo em estudos de melhor nível de evidência científica. O objetivo desta revisão narrativa é apresentar o que há de mais recente no tratamento tópico e sistêmico da acne vulgar. São enfatizadas as informações de revisões sistemáticas e de ensaios clínicos com bom desenho metodológico, obtidas a partir de busca sistematizada nas principais bases de dados em medicina. Há importantes questões referentes à conduta clínica diante da acne que ainda requerem embasamento em evidências científicas consistentes, entre elas a dose e a duração ideais do tratamento com antibióticos orais, sem que ocorra indução de resistência bacteriana, e a segurança da isotretinoína oral.

## INTRODUCTION

In cases of acne vulgaris, the initial therapeutic approach should take into account the severity of the clinical lesion type, i.e. whether there is predominance of inflammatory lesions or comedones. Classifying acne vulgaris according to the severity of the lesions into mild (papulopustular or comedonal), moderate (papulopustular or nodular) and severe (nodulocystic or conglobate) is a useful method for determining the most appropriate therapy, and this has been adopted in the treatment algorithms of the most recent international guidelines.^1^^,^^2^^,^^3^^,^^4^^,^^5^ Many widely practiced dermatological treatments lack support from studies providing high-level scientific evidence. The United States Institute of Medicine places comparative research into the effectiveness of acne treatment among the top 100 scientific priorities in that area in the country.^6^

## OBJECTIVES

The aim of this narrative review was to present the very latest good quality scientific evidence regarding topical and systemic treatments for acne vulgaris. Preference has been given to information from systematic reviews and well-designed, controlled and randomized clinical trials.

## METHODS

A systematic search was conducted in the major databases, for studies relating to treatment of acne between the years 2001 and 2011, using health descriptors or terms relating to the subject matter (Table 1).

**Table 1.** Systematic search for evidence on the treatment of acne vulgaris, performed on August 5, 2012

Database	Search strategy	Initial results	Studies selected for citation in the review	
			Systematic reviews	Randomized controlled trials
PubMed	“Acne Vulgaris” [Mesh]	2,915	17 (of 113)	6 (of 305)
Cochrane Library	“Acne Vulgaris” [Mesh]	737	8 (of 21)	6 (of 705)
Lilacs	mh: “acne vulgar”	271	0 (of 0)	0 (of 8)
Embase	‘acne vulgaris’/exp	2,064	9 (of 39)	6 (of 212)

## RESULTS AND DISCUSSION

In clinical practice, topical drugs have been shown to be effective for treating mild acne alone (except antibiotics) or in association with each other and/or with systemic medications. Several topical treatment options, with different modes of action, are available for acne. Although they are more effective than placebo in cases of mild acne, there is still no single initial or maintenance topical treatment strategy that is well validated by consistent scientific evidence.^3^^,^^4^^,^^5^^,^^6^

Benzoyl peroxide (BPO) is used for treating moderate and severe forms of acne alone or, preferably, in fixed or sequential combinations with topical retinoids, azelaic acid and topical or systemic antibiotics.^1^^,^^2^^,^^5^ It is also used for women with moderate to severe acne who are undergoing systemic hormonal anti-androgen therapy.^5^ It can be given as monotherapy at the outset of treatment for mild, papulopustular acne. In such cases, this treatment strategy appears as one of the first-line options in current British and German guidelines. The basis for this recommendation is the lower cost and longer track record of BPO regarding its safety and efficacy, in comparison with topical retinoids.^2^^,^^5^^,^^6^^,^^7^^,^^8^

BPO is an over-the-counter preparation. Its primary mechanism of action is antimicrobial activity, which reduces colonization of hair follicles by Propionibacterium acnes, while it also discreetly works to reduce inflammation and follicular hyperkeratinization.^5^^,^^6^

Unlike long-term antibiotic therapy, use of BPO does not induce bacterial resistance.^1^^,^^5^ Monotherapy with BPO has been shown to be as effective as an association of BPO with topical or oral antibiotics.^9^

In 2010, Seidler and Kimball conducted a meta-analysis in which they compared the efficacy of 5% BPO, 1% to 1.2% clindamycin, 5% BPO with salicylic acid, and a combination of BPO and clindamycin. The results showed that combinations of BPO were slightly more efficacious than use of this drug alone. While the difference was statistically significant, it is unlikely to be clinically important.^10^ Lower concentrations of BPO are recommended, as they produce less local irritation, which is the main adverse effect of medication.

Following a systematic review, Fakhouri et al. concluded that the efficacy of BPO is similar at concentrations of 2.5%, 5% and 10% and can be increased by addition of vitamin E and tertiary amines to the formulation, as well as by associating it with topical retinoids. According to these authors, new mechanisms for drug release have enhanced tolerability without decreasing efficacy.^8^^,^^11^

Retinoids are vitamin A derivatives that prevent formation of comedones by normalizing scaling of the follicular epithelium. They also have some anti-inflammatory action. Retinoids are contraindicated in pregnant women, and women with childbearing potential should use effective contraception simultaneously with treatment using these drugs. The main retinoids used topically for treating acne are: tretinoin, adapalene and tazarotene. The last of these is not currently available in Brazil.^6^^,^^12^

Topical retinoids are indicated for use alone in cases of mild acne (comedonal or papulopustular) and in maintenance treatment once the disease has been controlled. In moderate and severe forms, topical retinoids are used in fixed or sequential combinations with BPO, azelaic acid, and/or topical or systemic antibiotics. They are also recommended for women with moderate to severe acne who are undergoing systemic hormonal anti-androgen treatment.^5^

Several randomized controlled trials (RCTs) that compared topical retinoids with placebo have demonstrated their efficacy and tolerability. However, more studies are needed in order to compare the various topical retinoids with each other and in combinations with other drugs that are used to treat acne.^6^ In general, all topical retinoids are effective in reducing the number of comedones and inflammatory lesions by about 40% to 70%. Data from RCTs suggest that at higher concentrations, topical retinoids are more efficacious but provide a greater risk of irritation. Adapalene seems to have less potential for causing irritation and to be better tolerated than tretinoin and tazarotene, while tazarotene seems more efficacious.^12^ In 1998, Cunliffe et al. conducted a meta-analysis on five multicenter investigator-blinded RCTs, which compared the efficacy of 0.1% adapalene gel with 0.05% tretinoin gel and demonstrated that adapalene produces a better therapeutic response with lower incidence of skin irritation.^13^

A systematic review investigated whether the use of topical retinoids can cause temporary paradoxical worsening of lesions at baseline, which was a dogma hitherto widespread among North American dermatologists. The authors analyzed eight studies that found no evidence of worsening and only one study that suggested a slight deterioration of the clinical condition of acne during early treatment. They confirmed that topical retinoids cause skin irritation, but that good tolerance to the treatment develops over the first two to three months of treatment.^11^^,^^14^^,^^15^

The topical antibiotics most commonly used to treat acne are clindamycin and erythromycin. These improve acne by suppressing proliferation of Propionibacterium acne bacteria in the affected hair follicles, thus reducing local inflammation.^6^^,^^12^ In cases of acne, monotherapy with topical antibiotics is contraindicated due to the possibility of inducing bacterial resistance, which is related to worsened therapeutic responses and relatively slow onset of action compared with other antimicrobial regimens used in treating acne.^1^^,^^4^^,^^9^^,^^12^

In a systematic review carried out in 2008, Simonart and Draimax analyzed RCTs on therapeutic interventions for acne. They concluded that there had been a gradual decrease in efficacy of erythromycin over the previous years, which was probably related to the emergence of resistant strains of Propionibacterium acnes.^16^

Topical antibiotics, always in combination with topical retinoids, BPO or azelaic acid, are recommended for treatment of mild or moderate acne. They can also be used combined with systemic hormonal anti-androgen treatment in cases of moderate acne in women.^5^ Erythema, scaling, dryness and burning sensation may occur during treatment with topical antibiotics.^3^ Among the main contraindications and limitations to their use are pregnancy and breastfeeding for topical clindamycin and the risk of hepatotoxicity for erythromycin when applied to larger areas of skin among patients with abnormal liver function.^5^

Salicylic acid has been used for many years for treating acne and is present in several topical pharmaceutical preparations currently available for sale without prescription. Its action is exfoliative and comedolytic, but there is no consistent evidence that corroborates its routine use in preference to any other topical treatment. With regard to effectiveness, it is inferior to topical retinoids and is only recommended for patients who may not tolerate the irritation resulting from the action of retinoids.^4^^,^^6^

There are still some limitations in relation to the quality of the available evidence regarding the use of certain topical agents for management of acne, including sulfur, resorcinol, sodium sulfacetamide, aluminum chloride and zinc.^4^^,^^6^

Azelaic acid mainly presents comedolytic, antibacterial and mildly anti-inflammatory action, and its efficacy in acne treatment has been proven. The most common side effects are local irritation, erythema, pruritus, burning and mild flaking, which tend to disappear after four weeks of treatment.^17^^,^^18^^,^^19^ It can be indicated for the same clinical forms of acne and treatment conditions previously mentioned for topical retinoids, with the difference that use of azelaic acid does not present risks for pregnant and lactating women.^5^

There is evidence from RCTs that associations between two topical drugs with different mechanisms of action are more effective in resolving acne than monotherapy.^20^^,^^21^^,^^22^ Topical presentations that combine two anti-acne agents (e.g. topical retinoid or BPO + topical antibiotic, or topical retinoid + BPO) are recommended as the first choice for treating mild to moderate papular or papulopustular acne.^1^^,^^5^^,^^20^ Combinations of topical agents enhance treatment adherence due to their convenience of dosage, especially for single-use daily presentations, and their faster onset of action. Moreover, due to their greater efficacy compared with topical monotherapy, they also promote greater improvement in quality of life for patients with mild to moderate acne vulgaris.^22^^,^^23^^,^^24^^,^^25^

Oral medications for treating acne vulgaris are intended for use in moderate to severe cases or those that are refractory to topical medications.^6^^,^^11^^,^^13^ Oral antibiotics are among the agents of choice for severe cases of acne and moderate cases with inflammatory lesions affecting large areas of skin, especially the trunk. They can also be used in cases of mild to moderate inflammatory acne that fail to fully respond to topical therapy. They act by reducing bacterial colonization and inflammation in the affected follicles.^1^^,^^4^^,^^5^^,^^12^ Despite their effectiveness in reducing the number of inflammatory lesions, no oral antibiotic completely clears acne.^6^

Oral antibiotics should always be used in association with topical retinoids, BPO, azelaic acid and/or antiandrogenic medications, in the case of women, due to the possibility of developing bacterial resistance. This issue becomes more important when oral antibiotics are administered for long periods and/or at low doses. Concomitant use of topical and oral antibiotics is contraindicated.^1^^,^^5^^,^^26^^,^^27^

There is no conclusive evidence to show that any one antibiotic may be more effective than any other, even among the first and second generation tetracyclines that are the preferred oral antibiotic options for acne. However, among the tetracyclines, minocycline has greater potential for adverse effects, and has higher cost.^9^^,^^28^^,^^29^ The choice of oral antibiotic should be based on the patient’s preference, pattern of adverse events and cost.^6^

Combined oral contraceptives (COCs) suppress the activity of the sebaceous glands and reduce the formation of ovarian and adrenal androgens. Anti-androgen hormonal therapy with COCs should be used early in women with moderate to severe acne.^1^^,^^3^^,^^5^ There is good evidence, according to a Cochrane systematic review, that COCs do not differ much between each other with regard to their effectiveness in reducing inflammatory and non-inflammatory facial lesions. However, it is not yet possible to say to what degree COCs are effective in treating acne when compared with other treatments.^30^ There is no evidence to show that spironolactone is effective for treating acne, according to a Cochrane systematic review.^31^ None of the commercial preparations containing spironolactone currently available in Brazil or worldwide has been approved for use in treating acne. This is also the case with flutamide, which has been proscribed for treating acne as a result of its adverse effects, especially acute liver failure.^4^^,^^5^

Only oral isotretinoin has a mechanism of action that reaches all the pathogenic mechanisms relating to acne vulgaris. It is the most effective drug available against acne and promotes clinical cure in 85% of the cases. Isotretinoin is indicated in severe cases that are resistant to other therapies, and for patients prone to scarring or whose acne has a significant psychosocial impact.^1^^,^^5^ Consistent scientific evidence is still needed regarding its early use in mild cases, mainly because of the serious adverse effects related to isotretinoin. Such effects include teratogenicity and the possible risk of inflammatory bowel disease, depression and suicide, which have not been confirmed by population studies.^32^^,^^33^^,^^34^^,^^35^^,^^36^^,^^37^

There is good evidence in the literature that several treatments using light sources can improve inflammatory acne over the short term, and that photodynamic therapy provides results that are more consistent than other such therapies. However, there is no consistent evidence regarding the long-term therapeutic response or in relation to conventional acne treatments.^38^^,^^39^

Complementary and alternative treatments using herbs, aloe vera, pyridoxine, acids derived from fruits, tea tree oil, acupuncture and moxibustion are still limited to empirical use. Further well-conducted clinical trials are needed for the efficacy and safety of alternative therapies to be adequately defined.^6^^,^^40^

## CONCLUSION

A large number of commercial products are available for treating acne, among which several consist of combinations of different drugs. It is recognized that, so far, there are insufficient comparative studies to generate good quality evidence with regard to the treatments available for treating acne. This may explain the differences that exist among international guidelines regarding treatment recommendations and may explain also why they are not fully grounded in consistent scientific evidence. With regard to some aspects of acne treatment, the guidelines are limited to the opinions of experts, many of whom declare potentially important conflicts of interest.
